# Excess mortality in patients with non-functioning pituitary adenoma: a systematic review and meta-analysis

**DOI:** 10.1007/s40618-024-02356-9

**Published:** 2024-03-19

**Authors:** F. Bioletto, M. Sibilla, V. Gasco, E. Ghigo, S. Grottoli

**Affiliations:** Endocrinology, Diabetes and Metabolism, Department of Medical Sciences, University of Turin, Corso Dogliotti 14, Turin, 10126 Italy

**Keywords:** Non-functioning pituitary adenoma, NFPA, Standardized mortality ratio, SMR, Hypopituitarism

## Abstract

**Background:**

Patients with non-functioning pituitary adenoma (NFPA) often present with a variety of clinical manifestations and comorbidities, mainly determined by the local mass effect of the tumor and by hypopituitarism. Whether this has an impact on overall mortality, however, is still unclear.

**Methods:**

PubMed/Medline, EMBASE, and Cochrane Library databases were systematically searched until May 2023 for studies reporting data either about standardized mortality ratios (SMRs) or about predictors of mortality in patients with NFPA. Effect sizes were pooled through a random-effect model. This systematic review and meta-analysis was registered in the International Prospective Register of Systematic Reviews (PROSPERO, #CRD42023417782).

**Results:**

Eleven studies were eligible for inclusion in the systematic review; among these, five studies reported data on SMRs, with a total follow-up time of approximately 130,000 person-years. Patients with NFPA showed an increased mortality risk compared to the general population (SMR = 1.57 [95%CI: 1.20–1.99], p < 0.01). Age and sex appeared to act as effect modifiers, with a trend towards higher SMRs in females (SMR = 1.57 [95%CI: 0.91–2.41], p = 0.10) than in males (SMR = 1.00 [95%CI: 0.89–1.11], p = 0.97), and in patients diagnosed at age 40 years or younger (SMR = 3.19 [95%CI: 2.50–3.97], p < 0.01) compared to those with later onset of the disease (SMR = 1.26 [95%CI: 0.93–1.65], p = 0.13). The trend towards excess mortality was similar in patients with normal (SMR = 1.22 [95%CI: 0.94–1.53], p = 0.13) or deficient (SMR = 1.26 [95%CI: 0.82–1.79], p = 0.27) pituitary function.

**Conclusions:**

Excess mortality is observed in patients with NFPA, regardless of pituitary function, especially in women and in patients with a younger age at diagnosis.

**Supplementary Information:**

The online version contains supplementary material available at 10.1007/s40618-024-02356-9.

## Introduction

Non-functioning pituitary adenomas (NFPAs) are relatively common neoplasms arising from the adenohypophyseal cells [1], not associated with clinical or biochemical evidence of pituitary hormone hypersecretion [2]. NFPAs represent approximately 30% of all pituitary adenomas [3–5], with a yearly incidence of around 10 cases per million and a prevalence of 70–140 cases per million inhabitants [3, 5, 6].

Most of these tumors exhibit a benign behavior [1, 2, 7] and may be typically cured or controlled by surgery, radiotherapy, or even surveillance alone [8–11]. Nonetheless, they are frequently associated with various clinical manifestations and comorbidities, essentially determined by the local mass effect of the tumor on the surrounding tissues (e.g., headache, visual field defects, etc.) and to hypopituitarism [2, 9, 12]. Treatments for NFPAs might sometimes contribute to the onset of comorbidities [9], and patients treated for NFPA are overall characterized by an impaired quality of life [13].

Whether NFPAs are associated with an increased mortality compared with the general population is still a matter of debate. Studies assessing the long-term mortality outcomes of patients with NFPA are limited and provide conflicting results [14–19].

Given that patients with NFPA frequently have pituitary insufficiency at least to some degree, and that hypopituitarism of various etiologies is associated with increased mortality [20], a slightly increased mortality risk in patients with NFPA might be expected. However, this inference is not straightforward, as most studies demonstrating an increased mortality in patients with hypopituitarism were based on heterogeneous groups of patients with different underlying causes of hypopituitarism (e.g., functioning pituitary adenomas, craniopharyngiomas, etc.), having the potential to affect mortality regardless of pituitary hormone deficiencies [20–24]. Conversely, a slight mortality excess in NFPA patients with intact pituitary function cannot be excluded a priori, as some recent studies seem to point out [14, 15].

Other possible factors may also predict or account for mortality in patients with NFPA. Excess mortality has been shown to be higher in women than in men with pituitary disease [25], likely due to the fact that pituitary dysfunction jeopardizes the natural survival advantage that women have compared to men in the general population [25, 26]. However, it is still unclear whether this sexual dimorphism might be present also when focusing the analysis on patients with NFPA. Older age at diagnosis has been shown as an independent predictor of mortality in some series [14, 19, 27], although other studies suggest that, compared to the general population, excess mortality might be higher in younger patients [15, 16]. Radiotherapy (RT) may also contribute to an increased risk of premature death, mainly due to a higher incidence of cerebrovascular events [28].

Given this complex picture, the aim of this systematic review and meta-analysis was to evaluate all published data about mortality in patients with NFPA compared to the general population, as well as its possible independent predictors, in order to provide an up-to-date synthesis of the current knowledge in this regard.

## Methods

### Search strategy and study selection

This study was conducted according to the Preferred Reporting Items for Systematic Reviews and Meta-Analysis (PRISMA) guidelines [29]. The process of literature search and study selection was made by two independent reviewers (F.B., M.S.); all disparities were resolved through consensus. The following electronic databases were queried until May 6th 2023: PubMed/Medline, EMBASE, and Cochrane Library. The search strategy was performed using a combination of relevant database-specific search terms to identify studies evaluating mortality in patients with NFPA. The full search strategy is presented in the Supplementary Material (Appendix 1). No filters were applied for study design, language, and publication date. After duplicate removal, all studies found with the aforementioned search were evaluated for inclusion in the meta-analysis, first by title/abstract screening and then by full-text review. We excluded studies from our analysis according to the following exclusion criteria: (a) conference abstracts; (b) case reports or case series; (c) unavailability of any of the primary outcomes of interest, as defined in the following subsection. Study restricted to selected subgroups of patients with NFPA based on specific comorbidities or associated conditions (e.g., only patients with central adrenal insufficiency, only patients with GH deficiency, etc.) were not systematically searched and were excluded from the analysis. In case of patient overlap between two studies, the one with the largest patient cohort was considered; however, studies excluded from the main analysis for this reason could be still eligible for inclusion in subgroup analyses if they reported additional data that were not available in the main report. This systematic review and meta-analysis was registered in the International Prospective Register of Systematic Reviews (PROSPERO, #CRD42023417782).

### Outcomes

The primary outcomes of interest were: (i) the evaluation of standardized mortality ratio (SMR) in patients with NFPA; (ii) the evaluation of possible predictors of mortality in patients with NFPA. With respect to the first endpoint, stratified data according to sex, age at diagnosis (less or greater than 40 years old), and residual pituitary function were also collected, when provided.

### Data extraction

Two authors (F.B., M.S.) independently examined and extracted data from papers which met the inclusion criteria using pre-specified data extraction templates. For each eligible study, the following information were collected: (a) first author and publication year; (b) study design; (c) patient selection criteria; (d) number of subjects enrolled; (e) patients’ characteristics in terms of demographic data; (f) SMR in the whole NFPA cohort; (g) SMR in NFPA subgroups according to sex, age at diagnosis, and presence/absence of hypopituitarism; (h) internal predictors of mortality within study cohorts at survival analyses.

### Study quality and risk of bias assessment

The risk of bias was independently assessed for each included study by two authors (F.B., M.S.), according to the criteria described by Newcastle–Ottawa Scale [30]. This scale evaluates studies according to eight items belonging to three domains: selection (four items), comparability (one item) and outcome (three items). Each study can be awarded a maximum of one point for each item with the selection and outcome domains, and a maximum of two points for the item in the comparability domain. The overall quality of the studies is therefore evaluated on a 9-point scale, with scores of at least 5 being considered as indices of good quality.

### Statistical analysis

The SMR was chosen as the principal measure of outcome, computed by dividing the number of observed deaths by the number of expected deaths in a hypothetical cohort extracted from the general population and matched by age and sex to the patient group. The standard error (SE) of the SMR was calculated using the method by Vandenbroucke [31]. A random-effect model was adopted for the statistical pooling of data. Higgins I^2^ statistics and Cochran Q test were used to assess heterogeneity between studies. Small-study effects were evaluated by visual assessment of funnel plot asymmetry. A cut-off of 0.05 was adopted for the definition of statistical significance. Statistical analysis was performed using STATA 17 (StataCorp, College Station, Texas, USA).

## Results

### Search results and characteristics of the included studies

**Fig. 1:**
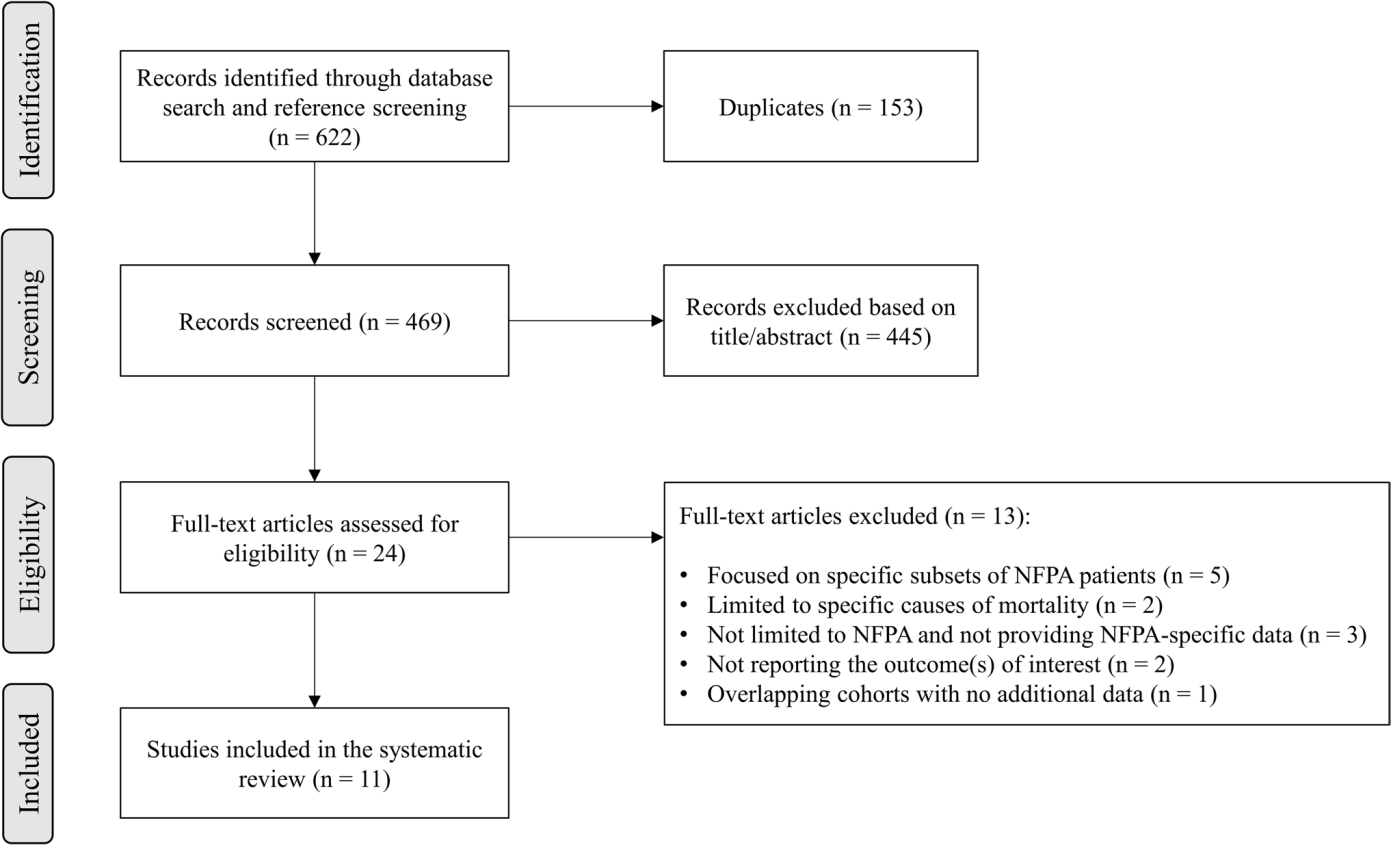
Flow-chart of study inclusion. Abbreviations: NFPA, non-functioning pituitary adenoma

A total of 622 records were identified in the initial literature search. Removal of duplicates led to an overall pool of 469 studies. An accurate title or abstract revision was sufficient to exclude 445 articles as not pertinent or not fulfilling our prespecified inclusion or exclusion criteria. The remaining 24 studies were assessed in full-text for eligibility; among these, 11 studies [14–19, 27, 32–35] met all criteria for being included in the final analysis (Fig. 1). Five studies [14–18] reported relevant data about the first outcome of interest (i.e., the evaluation of SMR in patients with NFPA), encompassing 23,123 patients with NFPA with a total follow-up time of approximately 130,000 person-years; one further study [32] reported data stratified by sex relative to the cohort of patients of one of the previous five [17]. Seven studies [14, 16, 19, 27, 33–35] reported relevant data about the second outcome of interest (i.e., the evaluation of predictors of mortality in patients with NFPA at internal analyses); two of them [34, 35] analyzed a subset of patients of a larger cohort [16], but were included in the qualitative synthesis of results because additional analyses of internal predictors were performed.

Table 1 summarizes the main study characteristics. All of them had a retrospective design. Three were single center studies [14, 18, 19], 3 were multicenter studies [17, 32, 33], and 5 were population-based studies [15, 16, 27, 34, 35]. In 4 studies [15, 16, 34, 35], the patient group was represented by patients treated or followed for NFPA, identified through the records of regional or national patient registries; in the other 7 studies [14, 17–19, 27, 32, 33], the patient group was represented only by patients who received active treatment for NFPA.

**Table 1:** Study characteristics. Abbreviations: HIRA-RID, health insurance review and assessment—rare intractable disease; N, number; NA, not available; NFPA, non-functioning pituitary adenoma; NHIRD, national health insurance research database; SMR, standardized mortality ratio

First author, year	Study design	Selection criteria of NFPA patients	N of subjects	Mean/ median follow-up (years)	Sex distribution (% male)	Mean/ median age (years**)**	Patients who received active treatment (%)	Patients with hypopituitarism (%)	Reporting SMR	Reporting internal analyses of predictors of mortality
Chang, 2008 [19]	Single center	Patients treated for NFPA	663	14.0	59	53.0	100	NA	No^a^	Yes
Dekkers, 2007 [18]	Single center	Patients treated for NFPA	174	9.1	56	55.3	100	91	Yes	No
Hammarstrand, 2017 [35]	Population-based	Patients with NFPA (followed in the western region of Sweden)	392	12.7	64	58.7	74	80	Yes^b^	Yes
Hsiao, 2019 [27]	Population-based	Patients treated for NFPA (registered in the Taiwan NHIRD database)	548	3.4^c^	58	43.4^c^	100	NA	No	Yes
Lindholm, 2006 [33]	Multicenter	Patients treated for NFPA	160	12.4	57	55.1^c^	100	70	Yes^d^	No
Nielsen, 2007 [17]	Multicenter	Patients treated for NFPA	192	10.8^c^	60	54.5^c^	100	73	Yes	No
Ntali, 2016 [14]	Single center	Patients treated for NFPA	546	8.0	61	58.7	100	NA	Yes	Yes
Oh, 2021 [15]	Population-based	Patients with NFPA (registered in HIRA-RID database)	19,416	5.3^e^	44	51.0	NA	21	Yes	No
Olsson, 2015 [16]	Population-based	Patients with NFPA (registered in the Swedish National Patients Registry)	2795	7.0	54	58.4	53	54	Yes	Yes
Olsson, 2017 [36]	Population-based	Patients with NFPA (followed in the western region of Sweden)	426	10.1^c^	64^c^	60.9^c^	72	NA	Yes^f^	Yes
O’Reilly, 2016 [34]	Multicenter	Patients treated for NFPA	519	7.0	62	57.0	100	83	No	Yes

^a^ SMR was reported to be significantly increased, but quantitative data were not specified

^b^ SMR data were not included in the meta-analysis because the patient cohort represented a subset of the one analysed by Olsson et al. (2015) [16]; however, this study was included in the evaluation of internal predictors of mortality, as it provided further relevant data compared to the study by Olsson et al. (2015) [16]

^c^ Pooled estimate

^d^ SMR data were not included in the main meta-analysis because the patient cohort represents a subset of the one analysed by Nielsen et al. (2007) [17]; however, this study was quantitatively included in subgroup analyses, as it provided additional relevant data stratified by presence/absence of hypopituitarism

^e^ Median follow-up in the whole cohort of patients with pituitary adenomas (functioning and non-functioning)

^f^ SMR data were not included in the meta-analysis because the patient cohort represented a subset of the one analysed by Olsson et al. (2015) [16]; however, this study was included in the evaluation of internal predictors of mortality, as it provided further relevant data compared to the study by Olsson et al. (2015) [16]

### Evaluation of standardized mortality ratio

**Fig. 2:**
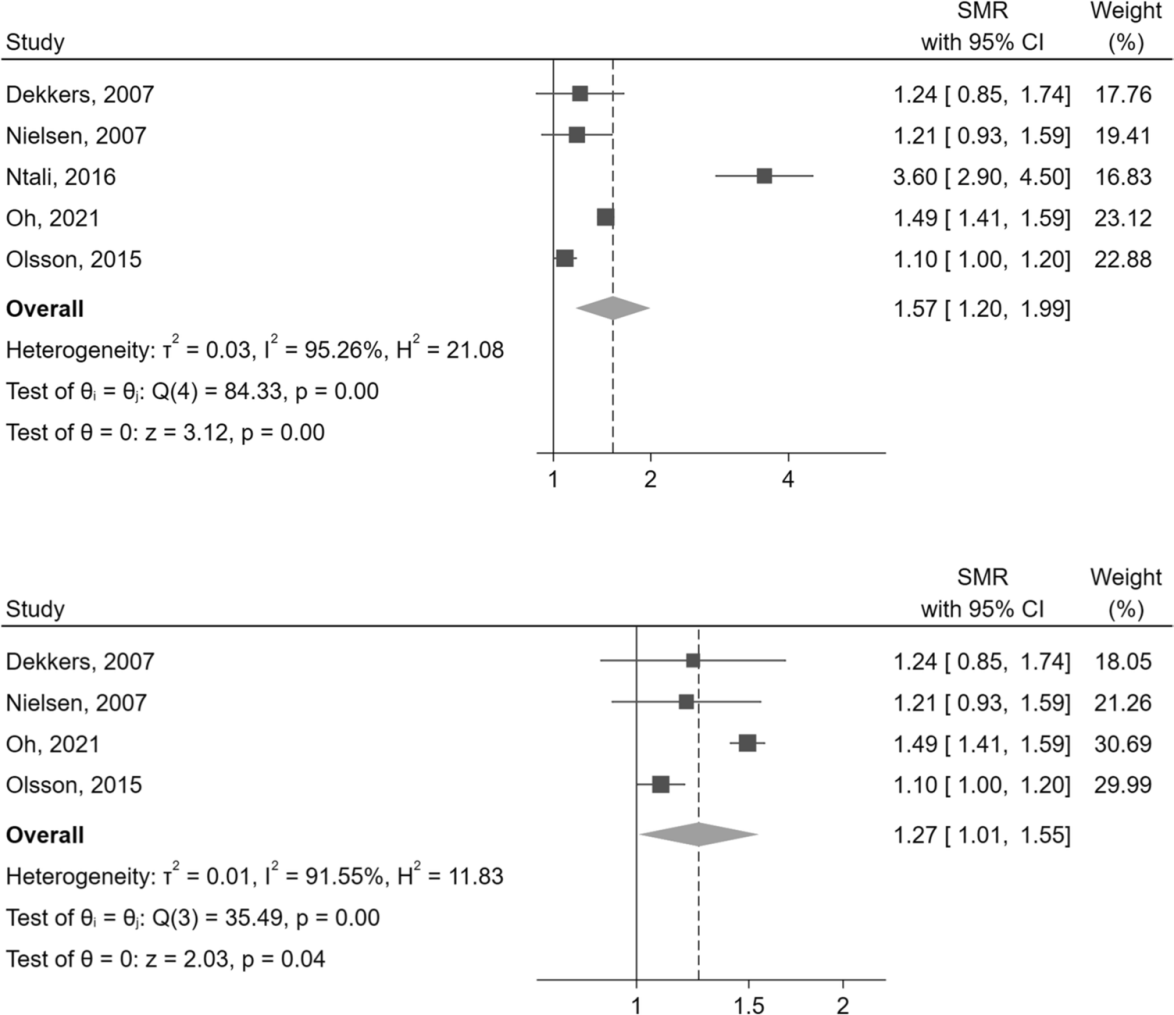
Mortality in NFPA compared to the general population, evaluated by SMR. The lower panel presents a sensitivity analysis after the exclusion of an outlier [14]. Abbreviations: CI, confidence interval; NFPA, non-functioning pituitary adenoma; SMR, standardized mortality ratio

Five studies [14–18] evaluating SMR in patients with NFPA fulfilled the pre-specified eligibility criteria. Overall, the SMR of patients with NFPA was significantly increased compared to the general population (SMR = 1.57 [95%CI: 1.20–1.99], p < 0.01), with a significant between-study heterogeneity (I^2^  =  95.26%, p < 0.01) (Fig. 2). One of these studies [14] was an outlier, with a SMR = 3.60 [95%CI: 2.90–4.50]. Even excluding this study in a sensitivity analysis, however, the pooled SMR remained significantly increased, though to a lesser extent (SMR = 1.27 [95%CI: 1.01–1.55], p = 0.04) (Fig. 2).

Three pre-specified subgroups analyses were performed by stratifying the SMR according to sex, age at diagnosis (less or greater than 40 years old) and to the presence/absence of hypopituitarism. Overall, two studies [16, 17] reported relevant data for the first subgroup analysis, two studies [15, 16] reported relevant data for the second subgroup analysis, and three studies [15, 16, 32] reported relevant data for the third one. Of note, one [32] of these three latter studies was not included in the main meta-analysis, because the patient cohort represented a subset of the one analysed by Nielsen et al. [17]; however, this study was quantitatively included in subgroup analyses, as it provided additional relevant data stratified by presence/absence of hypopituitarism.

**Fig. 3:**
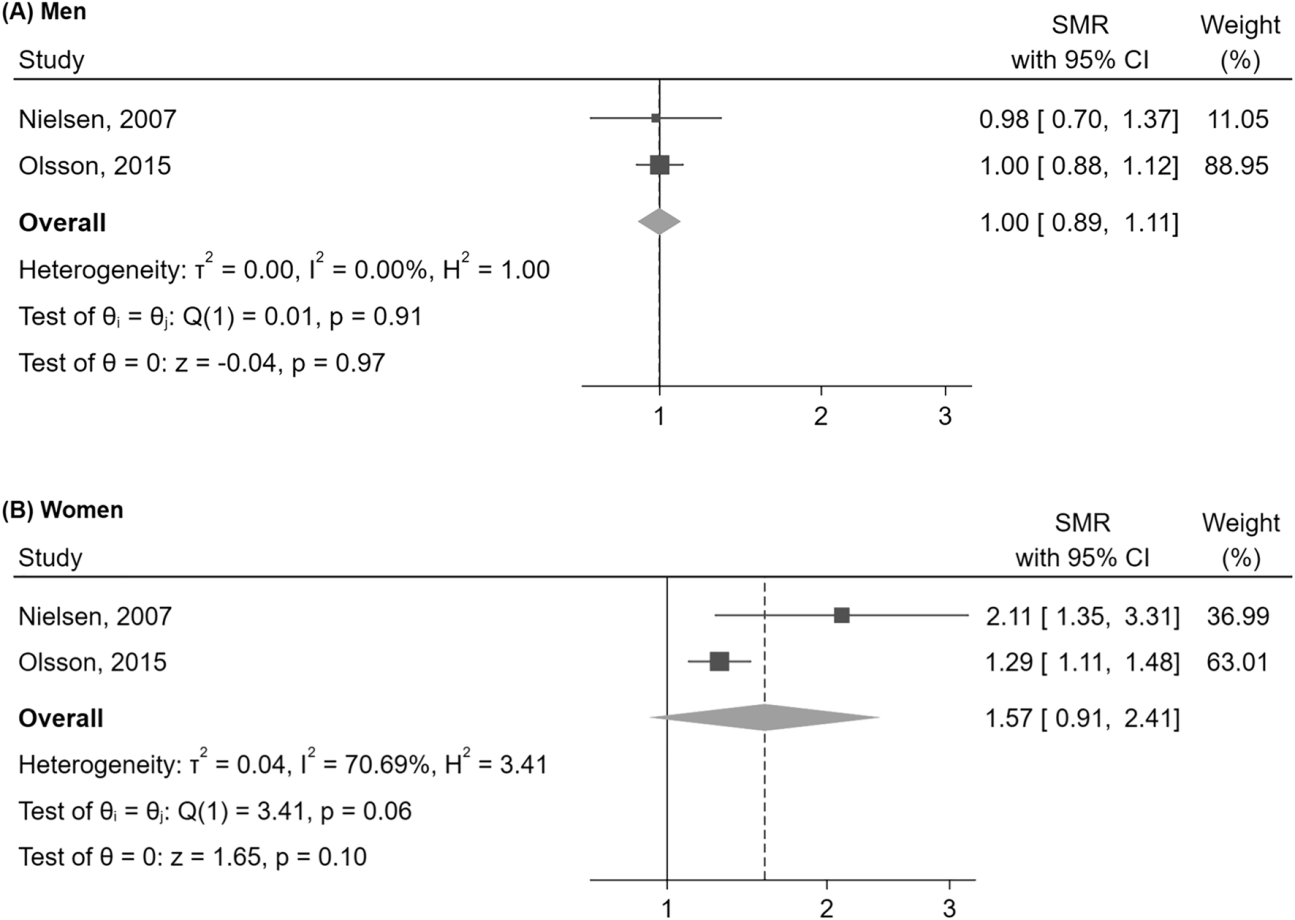
Mortality in NFPA compared to the general population, evaluated by SMR, stratified by sex. Abbreviations: CI, confidence interval; NFPA, non-functioning pituitary adenoma; SMR, standardized mortality ratio

Although limited by the small number of studies, the first subgroup analysis showed a possible higher mortality risk in women (SMR = 1.57 [95%CI: 0.91–2.41], p = 0.10) than in men (SMR = 1.00 [95%CI: 0.89–1.11], p = 0.97), though without reaching statistical significance in either subgroup (Fig. 3). Of note, the SMR in females was significantly increased in each of the two studies considered alone, with only a borderline-significant trend being maintained when pooling the data, as a consequence of the use of a random-effects model in a context of few studies with high between-study heterogeneity.

**Fig. 4:**
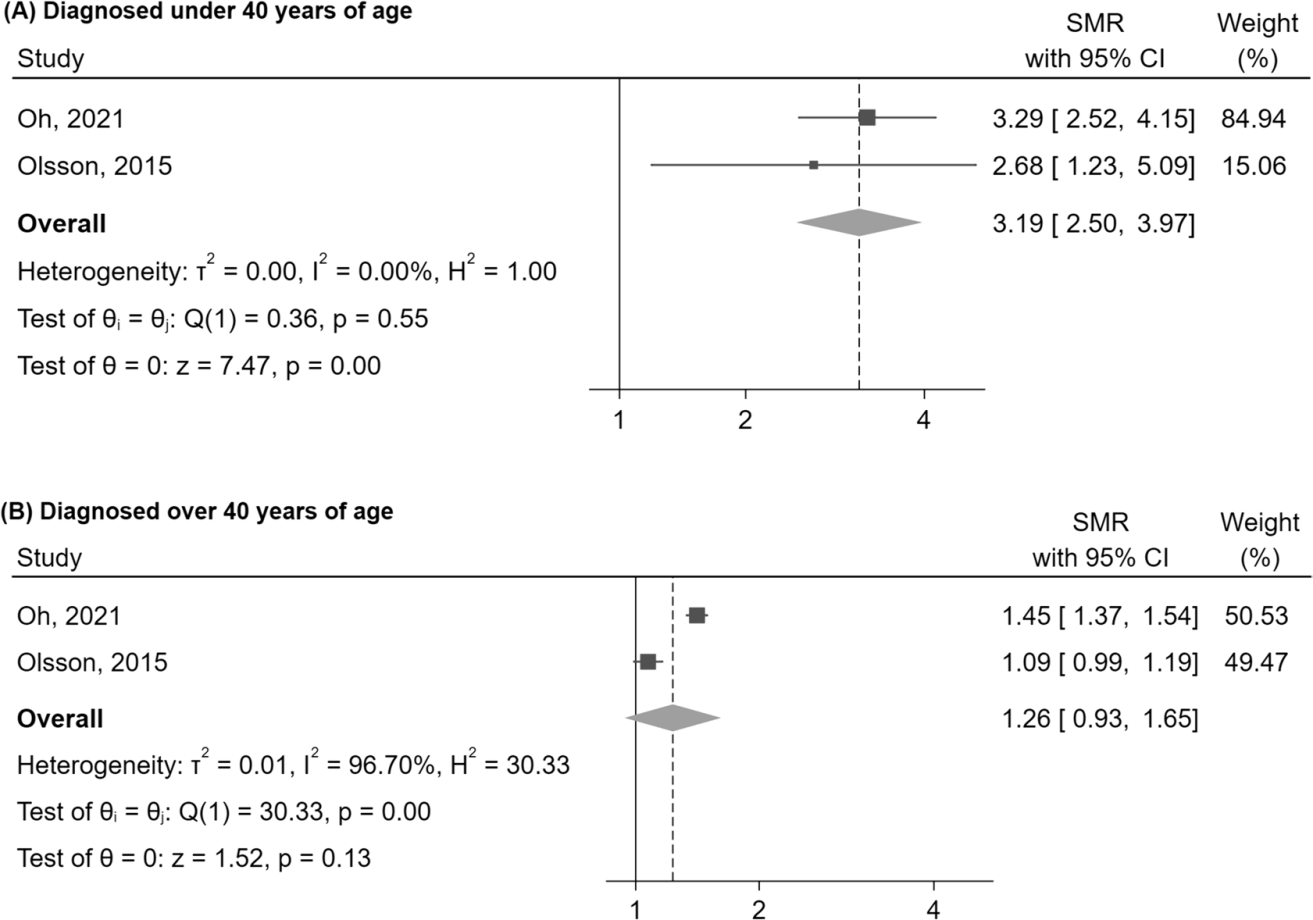
Mortality in NFPA compared to the general population, evaluated by SMR, stratified by age at diagnosis. Abbreviations: CI, confidence interval; NFPA, non-functioning pituitary adenoma; SMR, standardized mortality ratio

In the second subgroup analysis, the SMR was higher in patients diagnosed at less than 40 years of age (SMR = 3.19 [95%CI: 2.50–3.97], p < 0.01), than in those diagnosed afterwards (SMR = 1.26 [95%CI: 0.93–1.65], p = 0.13) (Fig. 4).

**Fig. 5:**
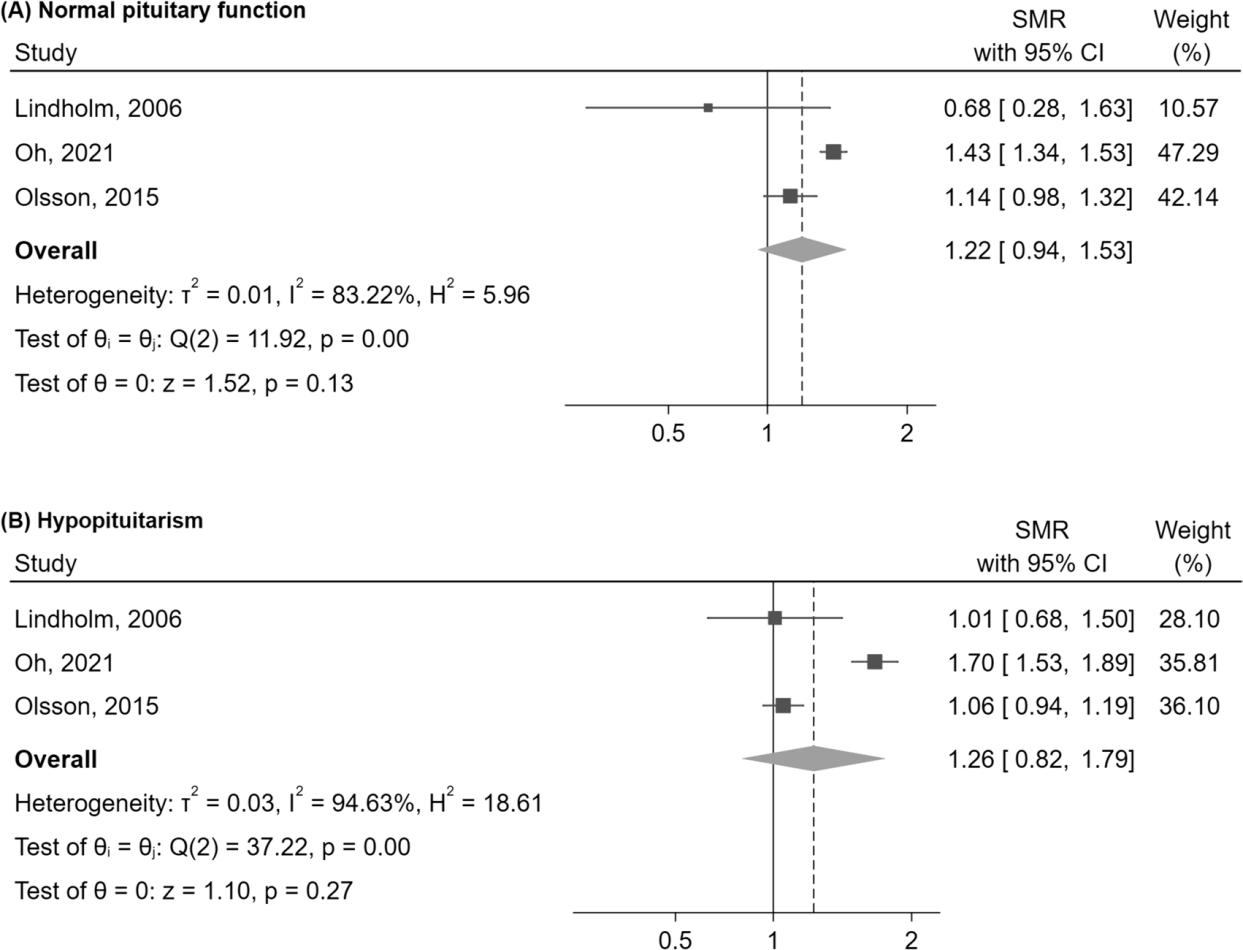
Mortality in NFPA compared to the general population, evaluated by SMR, stratified by pituitary function. Abbreviations: CI, confidence interval; NFPA, non-functioning pituitary adenoma; SMR, standardized mortality ratio

In the third subgroup analysis, the SMR was similar between patients with normal (SMR = 1.22 [95%CI: 0.94–1.53], p = 0.13) and deficient (SMR = 1.26 [95%CI: 0.82–1.79], p = 0.27) pituitary function, with a non-significant trend towards higher mortality in both subgroups (Fig. 5). Of note in this third analysis, it was not possible to quantitatively include the data from the study by Ntali et al. [14], as no specific stratification of results according to the presence or absence of hypopituitarism is provided; however, the authors provide a stratified analysis of SMRs according to the functional status of each single pituitary axis, finding an increase in mortality which was independent of their normal or deficient functioning. The inclusion of the results of this study, therefore, would have likely better supported the findings of an increased mortality in both subgroups.

### Evaluation of predictors of mortality at internal analyses

Seven studies [14, 16, 19, 27, 33–35] evaluated, within patients with NFPA, the possible role of various factors in the prediction of mortality. Any predictor evaluated in univariate or multivariable/adjusted survival analyses was considered and reported (Table 2). The results were characterized by an overall heterogeneity between studies, both in terms of the predictors that have been evaluated and of the quantitative findings that have been observed (Table 2).

**Table 2:** Predictors of mortality at internal analyses. Abbreviations: *ASA*, American Society of Anesthesiologists; *BMI*, body mass index; *CCI*, Charlson comorbidity index; *CI*, confidence interval; *F*, female; *GHD*, *GH* deficiency; *GHRT*, *GH* replacement therapy; *GTR*, gross-total resection; *HCeq*, hydrocortisone equivalent dose; *HR*, hazard ratio; *LT*4, levothyroxine; *M*, male; *STR*, subtotal resection

First author, year	Univariate analyses			Multivariable/adjusted analyses		
	Non-significant predictors	Significant predictors	HR (95%CI)	Non-significant predictors	Significant predictors	HR (95%CI)
Chang, 2008 [19]	Local extension Histopathological subtype	Age Size > 25 mm Male sex STR Radiotherapy	1.09 (1.08–1.17) 1.37 (1.05–1.79) 1.61 (1.25–2.08) 1.27 (1.01–1.62) 1.29 (1.00–1.67)	–	Age at diagnosis GTR and no radiotherapy	1.09 (1.08–1.84) 0.72 (0.54–0.94)
Hammarstrand, 2017 [35]	–	–	–	Sex^a^ Radiotherapy^a^	HCeq > 20 mg/day^a^	1.88 (1.06–3.33)
Hsiao, 2019 [27]	–	–	–	Transphenoidal surgery^b^ Sex Income Region of residence ASA scores	Stereotactic radiosurgery^b^ Age at diagnosis Charlson comorbidity index	0.36 (0.15–0.85) See note^c^ See note^d^
Ntali, 2016 [14]	Sex Presentation with apoplexy Cavernous sinus invasion Extent of surgical removal Repeat surgery for relapse Untreated hypogonadism ACTH deficiency TSH deficiency Diabetes insipidus	Age at diagnosis Radiotherapy Tumor regrowth Untreated GHD	(1.08–1.13) 2.21 (1.31–3.73) 1.99 (1.11–3.56) 0.32 (0.16–0.64)	Radiotherapy Tumor regrowth Untreated GHD	Age at diagnosis	1.10 (1.07–1.13)
Olsson, 2015 [16]	–	–	–	*Model 1* Hypopituitarism Diabetes insipidus	Age at diagnosis Diagnosed at ≤ 40 years	M: 1.12 (1.10–1.13) F: 1.13 (1.11–1.15) M: 3.47 (1.28–9.42) F: 4.20 (1.32–13.32)
				*Model 2* –	Age at diagnosis Radiotherapy	M: 1.11 (1.09–1.12) F: 1.12 (1.10–1.14) M: 1.99 (1.15–3.42) F: 2.81 (1.63–4.83)
Olsson, 2017 [36]	–	–	–	Radiotherapy BMI Sex Ongoing replacement therapies (other than GHRT)	Age at diagnosis Years of GHRT	1.10 (1.08–1.12) 0.94 (0.91–0.98)
O’Reilly, 2016 [34]	–	–	–	GHD^f^ TSH deficiency^f^ Diabetes insipidus^f^ Gonadal replacement therapy^g^	Radiotherapy^e^ Hypogonadism^f^ ACTH deficiency^f^ HCeq ≥ 30 mg/day^f^ LT4 < 100 µg/day^f^ Untreated GHD^f^	1.62 (1.01–2.60) 2.56 (1.10–5.96) 2.26 (1.15–4.47) 3.79 (1.49–9.67) 2.41 (1.23–4.73) 5.81 (0.73–46.23)^h^

^a^ The model was also adjusted for age, but no data was reported about the possible relationship between age and mortality

^b^ Compared to fractionated radiotherapy

^c^ Age was divided into 8 categories, with progressively increasing HR

^d^ Reference: CCI = 0; HR = 2.08 (1.33–3.26) for CCI = 1–2; HR = 4.56 (2.51–7.28) for CCI ≥ 3

^e^ Internal Cox adjusted for age at diagnosis, attained age, sex and surgery

^f^ Internal Cox adjusted for age at diagnosis, attained age, sex, surgery and radiotherapy

^g^ Internal Cox stratified by sex and adjusted for age at diagnosis, attained age, surgery and radiotherapy

^h^ This result is presented and discussed by the Authors as statistically significant (p = 0.04), although the reported 95%CI contains 1

When focusing on multivariable/adjusted analyses, age at diagnosis was found to be a positive predictor of mortality in four studies [14, 16, 19, 35], although in one of them [16] an independent increase in mortality risk was also observed in patients diagnosed at younger age (≤ 40 years). RT was independently associated with mortality in three studies [16, 19, 33], while no significant association was found in three others [14, 34, 35]; of note, in the study by Hsiao et al. [27], patients treated with stereotactic radiosurgery showed a lower risk of death as compared to those treated with fractionated RT. With regard to pituitary function, the findings varied between the different studies; hypopituitarism was not found to be a significant predictor of mortality neither by Ntali [14] nor by Olsson [16]; only in one study, by O’Reilly et al. [33], a significant association between some pituitary axis deficiencies (i.e., gonadotropin and ACTH deficiency) and mortality was found. Of note, in the same study [33], increased hydrocortisone doses (≥ 30 mg/day), lower levothyroxine doses (< 100 µg/day) and untreated GHD were associated with an increased risk of death. The finding of an increased mortality in patients replaced with higher glucocorticoid doses was also confirmed by Hammarstrand et al. [34]. The observation of a longer survival in patients on GH replacement therapy compared to those not treated with recombinant human GH (rhGH) was also made by Olsson et al. [35].

### Quality assessment and evaluation of small-study effects

The results of the quality assessment of the studies are reported in Supplementary Table 1 and 2. Altogether, the risk of bias appeared to be moderate-to-low in all studies. Visual assessment of funnel plot asymmetry revealed the possible presence of small-study effects due to the presence of an outlier (Supplementary Fig. 1); the potential impact of this on the results has already been addressed by specific sensitivity analysis.

## Discussion

This systematic review and meta-analysis showed that patients with NFPA have an increased risk of mortality compared to the general population. The pooled SMR was 1.57 (95%CI: 1.20–1.99) and remained significantly increased in a sensitivity analysis even after the exclusion of an outlier [14].

To the best of our knowledge, no previous meta-analyses had specifically evaluated mortality rates in patients with NFPA compared to the general population. The studies published to date reported heterogeneous results, showing either an increased [14, 15], borderline significant [16], or even normal rate of mortality [17, 18]. In the present meta-analysis, the statistical pooling of all published data shows that an increase in all-cause mortality in patients with NFPA is indeed present. Notably, this conclusion is further supported by the findings of a sixth study, by Chang et al. [19], which also reported a statistically significant increase in SMR for patients with NFPA, but could not be quantitatively pooled with the others because numerical data were not reported by the authors.

By definition, NFPAs are not associated with pituitary hormone hypersecretion [2], and are mostly benign in behaviour [1, 2, 7]. Nonetheless, they are frequently associated with relevant clinical manifestations due to the local mass effect of the tumor (e.g., headache, visual field defects) and/or to the effects of hypopituitarism [2, 9, 12]. Treatments for NFPAs might also contribute to the onset of comorbidities [9] and to an impairment in quality of life [13]. Whether this had an impact on overall mortality, however, was still unclear.

In the present study, we conducted three separate subgroup analyses, stratifying SMR according to sex, age at diagnosis, and presence or absence of hypopituitarism. The results of these analyses were limited by the small number of studies available, which made them likely underpowered, but their interpretation is still meaningful if properly taking this limitation into account.

Two studies [16, 17] examined separately the SMRs in males and females; in both cases, mortality was found to be significantly increased in women, while no excess mortality was observed in men. When pooling these data, the statistical significance of the increase in female mortality was formally lost, with only a borderline-significant trend being maintained, as a consequence of the use of a random-effect model in a context characterized by few studies with high between-study heterogeneity. However, based on the available data, it is reasonable to think that sex may indeed play a role as an effect modifier in this context, with excess mortality predominantly (if not uniquely) affecting females rather than males. This finding is not surprising, as an excess mortality in women has been already showed in other contexts of pituitary disease [25, 26]. The main hypothesis to justify this is that women with pituitary disease lose their natural survival advantage over men. Another possible explanation might be an underdiagnosis of hypopituitarism in women, since many of the diagnostic tests are not gender specific, with consequent undertreatment [25, 26]. Different effects of hormonal insufficiency and of replacement therapy between men and women are other factors that may also play a role [20, 25, 26].

When evaluating the role of age, two studies [15, 16] reported stratified SMRs data according to age at diagnosis; in both cases, a more marked increase in SMR was observed for patients diagnosed with NFPA at 40 years or younger, while excess mortality was less marked in patients older than that age. Notably, at internal analyses, older age was conversely found to be positively associated to mortality in few studies [14, 19, 27]. These two findings, however, are not necessarily in contrast; in fact, SMRs evaluates excess mortality compared to the general population, while internal analyses evaluate overall mortality within the cohort of NFPA patients. Not surprisingly, age acts as a positive predictor of overall mortality at internal analyses, as it does across the spectrum of most diseases. Conversely, when assessing excess mortality, younger patients exhibit the most evident disadvantage compared to the age- and sex-matched general population. Thus, similarly to sex, also age seems to act as an effect modifier, with excess mortality predominantly affecting younger patients rather than older ones.

When focusing on pituitary dysfunction, we found similar SMRs in patients with intact or deficient pituitary function, with a borderline-significant trend towards a higher mortality in both subgroups. At internal analyses, hypopituitarism was not found to be a significant predictor of mortality neither by Ntali [14] nor by Olsson [16]; only in the study by O’Reilly et al. [33] a significant association between some pituitary axis deficiencies (i.e., gonadotropin and ACTH deficiency) and mortality was found.

In previous studies, conducted in broader settings of patients with different pituitary diseases, hypopituitarism of various etiologies was shown to be associated with an increased mortality risk [20]. Inadequate hormonal replacement therapy has been proposed as a possible explanation of excess mortality [24, 26, 33, 36–38]. Replacement therapies have evolved and developed in the past decades, with the aim of being as close to normal physiology as possible, mimicking circadian rhythm and trying to avoid under/over treatment [39, 40]. Their impact on mortality in the setting of patients with NFPA has been investigated in some studies, as previously outlined. The available data suggest that GH replacement therapy is a safe option in patients with NFPA, with no evidence of increased mortality in treated patients. In the studies by Olsson et al. [35] and by O’Reilly et al. [33], treatment with rhGH was found to be associated with lower mortality; however, the observational nature of these finding makes it impossible to conclude towards a causal relationship. The finding of an association between a low LT4 replacement dose and mortality has been suggested as an isolate finding by O’Reilly et al. [33]; the interpretation of this result, however, is unclear; LT4 requirements are dependent on body weight, but this point was not accounted for in the analysis; moreover, monitoring parameters of under- or over-replacement (i.e., serum fT4 and fT3 values) were not reported; therefore, it is not possible to conclude whether the observed relationship between low replacement doses of LT4 and mortality was due to true under-replacement or to some unadjusted confounding. The relationship between higher doses of glucocorticoid replacement therapy and increased mortality is more consistent. Two studies [33, 34] examined the relationship between glucocorticoid replacement dose and mortality, and in both cases a significant association between higher doses and an increased risk of death was observed. This is in line with findings in other contexts [36, 37, 41], in which higher glucocorticoid replacement doses were associated with an adverse cardiometabolic profile, and with a consequent increase in morbidity and mortality. Nevertheless, it should be noted that this association does not necessarily imply a causal relationship, as the available evidence is observational in nature and, thus, selection bias or other systematic errors cannot be completely ruled out.

Based on the available evidence, a slight mortality excess also in NFPA patients with apparently preserved pituitary function cannot be excluded. As previously outlined, we did not find consistent evidence supporting a difference in excess mortality in patients with NFPA with or without hypopituitarism, except possibly in case of inadequate hormonal replacement therapies. Notably, in the study by Oh et al. [15], which is the largest currently available, patients with normal pituitary function were found to have a significantly increased mortality. In the study by Olsson et al. [16], a slight borderline-significant increase in mortality was also found. Ntali et al. [14] did not report data based on preserved or deficient pituitary function as a whole, but they evaluated deficits in each single axis individually and, interestingly, an increased mortality was found also in patients with intact tropin secretions.

The reason behind these results is not clear. In some cases, health consequences due to local mass effect and/or to the treatments received might contribute to mortality regardless of their effect on pituitary function [2, 8]. In other cases, an underdiagnosis of hypopituitarism might be hypothesized. For instance, patients that are not candidate for GH replacement therapy might not be tested for GHD, which may thus remain undiagnosed in some cases [42, 43]. More in general, however, it cannot be excluded that NFPA patients with apparently preserved pituitary function may harbor subclinical alterations in the mechanisms of control of pituitary secretion compared to a completely healthy hypophysis, which do not get detected by the commonly used diagnostic work-up. In fact, the diagnosis of hypopituitarism essentially relies on standardized tests and reference values, taking little account of individual hormonal setpoints [26, 44] and potentially leading to an under-recognition of milder functional derangements. To give an example, although the general population may exhibit a wider spectrum of TSH and fT4 values, individual TSH/fT4 levels tend to remain relatively stable over time [26, 45]; consequently, patients having their natural “thyrostat” set to mid-high fT4 levels might still maintain fT4 levels within the normal range even when a mild damage of their hypothalamus–pituitary–thyroid axis is present [26]; similar arguments can also be made with regard to other axes, and underline the limitations of current diagnostic tools for hypopituitarism. Clearly, even accepting the hypothesis that some alterations of pituitary secretion might in part go undiagnosed using standard references, the clinical implications are still unclear; on the one hand, it might be argued that only milder derangements are missed by standard diagnostic tests; however, though being mild, they remain untreated, and this might still determine a slight excess in mortality when large cohorts are analyzed. Nevertheless, further studies are needed to analyze this aspect and its possible clinical significance.

With regard to treatments, in particular, the role of RT as an independent predictor of mortality has been investigated by several authors. Among the studies included in this systematic review, RT was associated with mortality in three of them [16, 19, 33], while three others [14, 34, 35] found no differences between irradiated and non-irradiated patients. Of note, among irradiated patients, possible differences between different RT protocols (i.e., radiosurgery vs fractionated RT) might be present [27]. RT-induced hypopituitarism is a relatively frequent consequence of RT [26] and can at least in part explain an excess in morbidity and mortality in irradiated patients; nevertheless, a direct detrimental effect of RT can also be present, mainly due to a higher incidence of cerebrovascular events [28].

Our analysis had some limitations. Firstly, the strength of the conclusions was limited by the low number of available studies; this was particularly true for the subgroup analyses, which were likely underpowered to properly evaluate possible differences in the assessment of the outcome. Secondly, the quality was limited by that of the included studies; nevertheless, according to the Newcastle–Ottawa scale, the risk of bias was generally moderate-to-low, which reassured about the likely small impact of this issue on the final results. Thirdly, patients’ inclusion criteria were partly different between studies, and this could be responsible for a certain degree of heterogeneity in the reported results; however, heterogeneity is a common limitation of all meta-analyses, and appropriate statistical methods – such as the use of a random-effect model – were adopted to account for it. Fourthly, the available evidence comes from patients treated for NFPA or registered for NFPA in national disease registries; therefore, it is essentially representative of lesions considered to be of actual or potential clinical relevance. Consequently, the results obtained should not be generalized to patients with small non-functioning microadenomas, which usually do not cause any signs or symptoms, are often discovered incidentally, do not need any active treatment in the majority of cases, and are most likely completely benign entities with no impact on life expectancy, morbidity or quality of life.

In conclusion, our study was the first meta-analysis evaluating the impact of NFPAs on mortality. A slight excess in mortality could be observed, and was more evident for women and for younger patients (< 40 years old). On the other hand, patients with intact or deficient pituitary function had comparable SMRs, with a non-significant trend towards higher mortality in both subgroups.

### Supplementary Information

Below is the link to the electronic supplementary material.

Supplementary file1 (DOCX 80 KB)

## Data Availability

Not applicable.
