# Supplementary material for: Excess mortality in patients with non-functioning pituitary adenoma: a systematic review and meta-analysis
Source: J Endocrinol Invest. 2024 Mar 19;47(9):2143–55. doi: 10.1007/s40618-024-02356-9 (PMC11369010; doi:10.1007/s40618-024-02356-9)
Supplement: Supplementary file 1 — Supplementary file1 (DOCX 80 KB) [file 40618_2024_2356_MOESM1_ESM.docx]

**Supplementary Material**

**Supplementary Table 1.** Risk of bias assessment with regard to SMR analyses, according to Newcastle-Ottawa scale. Abbreviations: SMR, standardized mortality ratio.

| **First author,**  **year** | **Selection** | | | | **Comparability** | **Outcome** | | | **Total score** |
| --- | --- | --- | --- | --- | --- | --- | --- | --- | --- |
|  | **Representativeness of Exposed Cohort** | **Selection of Nonexposed Cohort** | **Ascertainment of Exposure** | **Outcome Not Present at Baseline** | **Comparability of cohorts** | **Assessment of Outcome** | **Sufficient Follow-Up Duration** | **Adequate Follow-up** |  |
| Dekkers,  2007 (18) | 1 | 1 | 1 | 1 | 2 | 1 | 1 | 1 | 9 |
| Hammarstrand,  2017 (35) | 1 | 1 | 1 | 1 | 2 | 1 | 1 | 1 | 9 |
| Lindholm,  2006 (33) | 1 | 1 | 1 | 1 | 2 | 1 | 1 | 1 | 9 |
| Nielsen,  2007 (17) | 1 | 1 | 1 | 1 | 2 | 1 | 1 | 1 | 9 |
| Ntali,  2016 (14) | 1 | 1 | 1 | 1 | 2 | 1 | 1 | 1 | 9 |
| Oh,  2021 (15) | 1 | 1 | 0 | 1 | 2 | 1 | 1 | 1 | 8 |
| Olsson,  2015 (16) | 1 | 1 | 0 | 1 | 2 | 1 | 1 | 1 | 8 |
| Olsson,  2017 (36) | 1 | 1 | 1 | 1 | 2 | 1 | 1 | 1 | 9 |

**Supplementary Table 2.** Risk of bias assessment with regard to internal analyses of predictors of mortality, according to Newcastle-Ottawa scale.

| **First author,**  **year** | **Selection** | | | | **Comparability** | **Outcome** | | | **Total score** |
| --- | --- | --- | --- | --- | --- | --- | --- | --- | --- |
|  | **Representativeness of Exposed Cohort** | **Selection of Nonexposed Cohort** | **Ascertainment of Exposure** | **Outcome Not Present at Baseline** | **Comparability of cohorts** | **Assessment of Outcome** | **Sufficient Follow-Up Duration** | **Adequate Follow-up** |  |
| Chang,  2008 (19) | 1 | 1 | 1 | 1 | 1 | 1 | 1 | 1 | 8 |
| Hammarstrand,  2017 (35) | 1 | 1 | 1 | 1 | 1 | 1 | 1 | 1 | 8 |
| Hsiao,  2019 (27) | 1 | 1 | 0 | 1 | 1 | 1 | 1 | 1 | 7 |
| Ntali,  2016 (14) | 1 | 1 | 1 | 1 | 1 | 1 | 1 | 1 | 8 |
| Olsson,  2015 (16) | 1 | 1 | 0 | 1 | 2 | 1 | 1 | 1 | 8 |
| Olsson,  2017 (36) | 1 | 1 | 1 | 1 | 2 | 1 | 1 | 1 | 9 |
| O’Reilly,  2016 (34) | 1 | 1 | 1 | 1 | 2 | 1 | 1 | 1 | 9 |

**Supplementary Figure 1.** Evaluation of small-study effects by visual assessment of funnel plot asymmetry. Effect sizes and standard errors are reported on a square root scale, according to the method by Vandenbroucke (31). Abbreviations: CI, confidence interval; sqrt(SMR), square root of the standardized mortality ratio.


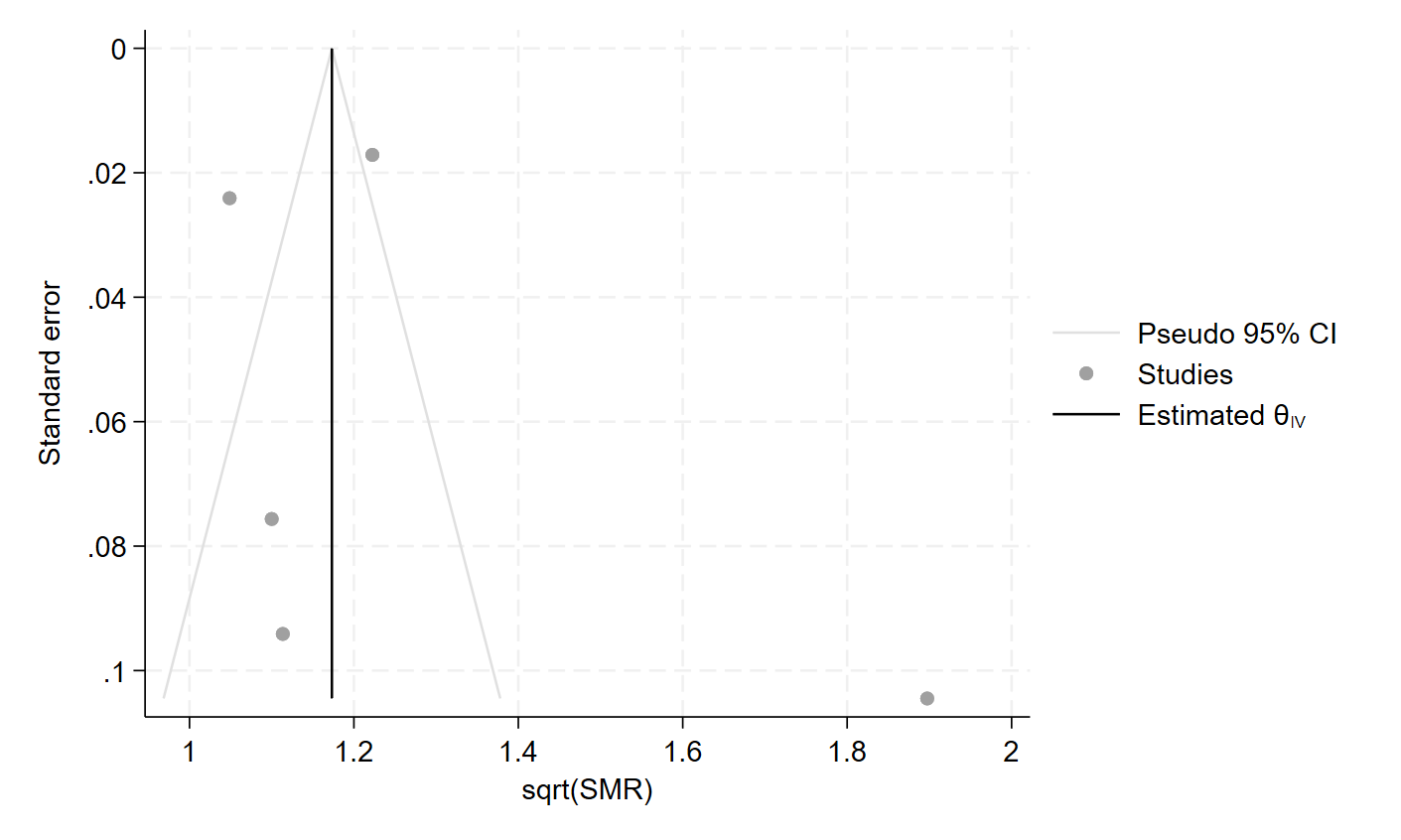


**Appendix 1.** Electronic search strategy

| **PUBMED** |
| --- |
| *Advanced search, All fields* |
| (  (“non-functioning pituitary adenoma” OR “non-functioning pituitary adenomas” OR “nonfunctioning pituitary adenoma” OR “nonfunctioning pituitary adenomas” OR “non-functional pituitary adenoma” OR “non-functional pituitary adenomas” OR “nonfunctional pituitary adenoma” OR “nonfunctional pituitary adenomas”)  OR  (“non-functioning pituitary microadenoma” OR “non-functioning pituitary microadenomas” OR “nonfunctioning pituitary microadenoma” OR “nonfunctioning pituitary microadenomas” OR “non-functional pituitary microadenoma” OR “non-functional pituitary microadenomas” OR “nonfunctional pituitary microadenoma” OR “nonfunctional pituitary microadenomas”)  OR  (“non-functioning pituitary macroadenoma” OR “non-functioning pituitary macroadenomas” OR “nonfunctioning pituitary macroadenoma” OR “nonfunctioning pituitary macroadenomas” OR “non-functional pituitary macroadenoma” OR “non-functional pituitary macroadenomas” OR “nonfunctional pituitary macroadenoma” OR “nonfunctional pituitary macroadenomas”)  OR  (“non-functioning pituitary tumor” OR “non-functioning pituitary tumors” OR “nonfunctioning pituitary tumor” OR “nonfunctioning pituitary tumors” OR “non-functional pituitary tumor” OR “non-functional pituitary tumors” OR “nonfunctional pituitary tumor” OR “nonfunctional pituitary tumors”)  OR  (“pituitary” AND (“non-functioning adenoma” OR “non-functioning adenomas” OR “nonfunctioning adenoma” OR “nonfunctioning adenomas” OR “non-functional adenoma” OR “non-functional adenomas” OR “nonfunctional adenoma” OR “nonfunctional adenomas”))  OR  (“pituitary” AND (“non-functioning microadenoma” OR “non-functioning microadenomas” OR “nonfunctioning microadenoma” OR “nonfunctioning microadenomas” OR “non-functional microadenoma” OR “non-functional microadenomas” OR “nonfunctional microadenoma” OR “nonfunctional microadenomas”))  OR  (“pituitary” AND (“non-functioning macroadenoma” OR “non-functioning macroadenomas” OR “nonfunctioning macroadenoma” OR “nonfunctioning macroadenomas” OR “non-functional macroadenoma” OR “non-functional macroadenomas” OR “nonfunctional macroadenoma” OR “nonfunctional macroadenomas”))  OR  (“pituitary” AND (“non-functioning tumor” OR “non-functioning tumors” OR “nonfunctioning tumor” OR “nonfunctioning tumors” OR “non-functional tumor” OR “non-functional tumors” OR “nonfunctional tumor” OR “nonfunctional tumors”))  )  AND  (“mortality” OR “death” OR “survival”) |

| **EMBASE** |
| --- |
| *Quick search, All fields* |
| (  (“non-functioning pituitary adenoma” OR “non-functioning pituitary adenomas” OR “nonfunctioning pituitary adenoma” OR “nonfunctioning pituitary adenomas” OR “non-functional pituitary adenoma” OR “non-functional pituitary adenomas” OR “nonfunctional pituitary adenoma” OR “nonfunctional pituitary adenomas”)  OR  (“non-functioning pituitary microadenoma” OR “non-functioning pituitary microadenomas” OR “nonfunctioning pituitary microadenoma” OR “nonfunctioning pituitary microadenomas” OR “non-functional pituitary microadenoma” OR “non-functional pituitary microadenomas” OR “nonfunctional pituitary microadenoma” OR “nonfunctional pituitary microadenomas”)  OR  (“non-functioning pituitary macroadenoma” OR “non-functioning pituitary macroadenomas” OR “nonfunctioning pituitary macroadenoma” OR “nonfunctioning pituitary macroadenomas” OR “non-functional pituitary macroadenoma” OR “non-functional pituitary macroadenomas” OR “nonfunctional pituitary macroadenoma” OR “nonfunctional pituitary macroadenomas”)  OR  (“non-functioning pituitary tumor” OR “non-functioning pituitary tumors” OR “nonfunctioning pituitary tumor” OR “nonfunctioning pituitary tumors” OR “non-functional pituitary tumor” OR “non-functional pituitary tumors” OR “nonfunctional pituitary tumor” OR “nonfunctional pituitary tumors”)  OR  (“pituitary” AND (“non-functioning adenoma” OR “non-functioning adenomas” OR “nonfunctioning adenoma” OR “nonfunctioning adenomas” OR “non-functional adenoma” OR “non-functional adenomas” OR “nonfunctional adenoma” OR “nonfunctional adenomas”))  OR  (“pituitary” AND (“non-functioning microadenoma” OR “non-functioning microadenomas” OR “nonfunctioning microadenoma” OR “nonfunctioning microadenomas” OR “non-functional microadenoma” OR “non-functional microadenomas” OR “nonfunctional microadenoma” OR “nonfunctional microadenomas”))  OR  (“pituitary” AND (“non-functioning macroadenoma” OR “non-functioning macroadenomas” OR “nonfunctioning macroadenoma” OR “nonfunctioning macroadenomas” OR “non-functional macroadenoma” OR “non-functional macroadenomas” OR “nonfunctional macroadenoma” OR “nonfunctional macroadenomas”))  OR  (“pituitary” AND (“non-functioning tumor” OR “non-functioning tumors” OR “nonfunctioning tumor” OR “nonfunctioning tumors” OR “non-functional tumor” OR “non-functional tumors” OR “nonfunctional tumor” OR “nonfunctional tumors”))  )  AND  (“mortality” OR “death” OR “survival”)  NOT  (conference review OR conference abstract).pt |

| **COCHRANE LIBRARY** |
| --- |
| *Advanced search, All text* |
| (  (“non-functioning pituitary adenoma” OR “non-functioning pituitary adenomas” OR “nonfunctioning pituitary adenoma” OR “nonfunctioning pituitary adenomas” OR “non-functional pituitary adenoma” OR “non-functional pituitary adenomas” OR “nonfunctional pituitary adenoma” OR “nonfunctional pituitary adenomas”)  OR  (“non-functioning pituitary microadenoma” OR “non-functioning pituitary microadenomas” OR “nonfunctioning pituitary microadenoma” OR “nonfunctioning pituitary microadenomas” OR “non-functional pituitary microadenoma” OR “non-functional pituitary microadenomas” OR “nonfunctional pituitary microadenoma” OR “nonfunctional pituitary microadenomas”)  OR  (“non-functioning pituitary macroadenoma” OR “non-functioning pituitary macroadenomas” OR “nonfunctioning pituitary macroadenoma” OR “nonfunctioning pituitary macroadenomas” OR “non-functional pituitary macroadenoma” OR “non-functional pituitary macroadenomas” OR “nonfunctional pituitary macroadenoma” OR “nonfunctional pituitary macroadenomas”)  OR  (“non-functioning pituitary tumor” OR “non-functioning pituitary tumors” OR “nonfunctioning pituitary tumor” OR “nonfunctioning pituitary tumors” OR “non-functional pituitary tumor” OR “non-functional pituitary tumors” OR “nonfunctional pituitary tumor” OR “nonfunctional pituitary tumors”)  OR  (“pituitary” AND (“non-functioning adenoma” OR “non-functioning adenomas” OR “nonfunctioning adenoma” OR “nonfunctioning adenomas” OR “non-functional adenoma” OR “non-functional adenomas” OR “nonfunctional adenoma” OR “nonfunctional adenomas”))  OR  (“pituitary” AND (“non-functioning microadenoma” OR “non-functioning microadenomas” OR “nonfunctioning microadenoma” OR “nonfunctioning microadenomas” OR “non-functional microadenoma” OR “non-functional microadenomas” OR “nonfunctional microadenoma” OR “nonfunctional microadenomas”))  OR  (“pituitary” AND (“non-functioning macroadenoma” OR “non-functioning macroadenomas” OR “nonfunctioning macroadenoma” OR “nonfunctioning macroadenomas” OR “non-functional macroadenoma” OR “non-functional macroadenomas” OR “nonfunctional macroadenoma” OR “nonfunctional macroadenomas”))  OR  (“pituitary” AND (“non-functioning tumor” OR “non-functioning tumors” OR “nonfunctioning tumor” OR “nonfunctioning tumors” OR “non-functional tumor” OR “non-functional tumors” OR “nonfunctional tumor” OR “nonfunctional tumors”))  )  AND  (“mortality” OR “death” OR “survival”) |
